# Speed or Accuracy Instructions During Skill Learning do not Affect the Acquired Knowledge

**DOI:** 10.1093/texcom/tgaa041

**Published:** 2020-08-10

**Authors:** Teodóra Vékony, Hanna Marossy, Anita Must, László Vécsei, Karolina Janacsek, Dezso Nemeth

**Affiliations:** Department of Neurology, University of Szeged, 6725 Szeged, Hungary; Institute of Psychology, ELTE Eötvös Loránd University, 1064 Budapest, Hungary; Institute of Psychology, University of Szeged, 6722 Szeged, Hungary; Department of Neurology, University of Szeged, 6725 Szeged, Hungary; MTA-SZTE Neuroscience Research Group, University of Szeged, 6725 Szeged, Hungary; Institute of Psychology, ELTE Eötvös Loránd University, 1064 Budapest, Hungary; Brain, Memory and Language Research Group, Institute of Cognitive Neuroscience and Psychology, Research Centre for Natural Sciences, 1117 Budapest, Hungary; Centre for Thinking and Learning, Institute for Lifecourse Development, School of Human Sciences, Faculty of Education, Health and Human Sciences, University of Greenwich, Old Royal Naval College, London, SE10 9LS UK; Institute of Psychology, ELTE Eötvös Loránd University, 1064 Budapest, Hungary; Brain, Memory and Language Research Group, Institute of Cognitive Neuroscience and Psychology, Research Centre for Natural Sciences, 1117 Budapest, Hungary; Lyon Neuroscience Research Center (CRNL), INSERM, CNRS, Université Claude Bernard Lyon 1, 69675 Bron, France

**Keywords:** implicit learning, instruction, probabilistic learning, speed-accuracy, statistical learning

## Abstract

A crucial question in skill learning research is how instruction affects the performance or the underlying representations. Little is known about the effects of instructions on one critical aspect of skill learning, namely, picking-up statistical regularities. More specifically, the present study tests how prelearning speed or accuracy instructions affect the acquisition of non-adjacent second-order dependencies. We trained 2 groups of participants on an implicit probabilistic sequence learning task: one group focused on being fast and the other on being accurate. As expected, we detected a strong instruction effect: accuracy instruction resulted in a nearly errorless performance, and speed instruction caused short reaction times (RTs). Despite the differences in the average RTs and accuracy scores, we found a similar level of statistical learning performance in the training phase. After the training phase, we tested the 2 groups under the same instruction (focusing on both speed and accuracy), and they showed comparable performance, suggesting a similar level of underlying statistical representations. Our findings support that skill learning can result in robust representations, and they highlight that this form of knowledge may appear with almost errorless performance. Moreover, multiple sessions with different instructions enabled the separation of competence from performance.

## Introduction

Our social, motor, and cognitive skills help us adapt to and function in various situations in our everyday life. Therefore, fine-tuning the ability to learn new skills can be advantageous for an individual. Previous studies investigating sports performance (Beilock et al. 2004, 2008) and sequence learning (Hoyndorf and Haider 2009; Barnhoorn et al. 2019) found that speed and accuracy strategies differently affect skill learning. However, skill learning is multifaceted, and it is still not clear what underlying mechanisms benefit from speed and accuracy instructions and what mechanisms do not. A core component of learning new skills is picking up complex statistical regularities from the environment (Janacsek et al. 2012; Conway 2020). To date, no study has investigated the effects of prioritizing speed or accuracy on the acquisition of such statistical dependencies. Here, we aim to unveil how emphasizing speed or accuracy influences this essential aspect of skill learning.

Hoyndorf and Haider (2009) investigated the sequencing aspect of skill learning and found an accuracy strategy to impair the expression of implicit knowledge compared to speed instruction; however, evidence of learning was still detected under accuracy instruction compared to a non-learning control group. Yet, in this experiment, the accumulated sequence-knowledge under speed/accuracy instructions was not compared to a phase where the importance of speed and accuracy was equally emphasized. Such a comparison would reveal whether implicit sequence knowledge is acquired at the same level under different instructions. Recently, Barnhoorn et al. (2019) found that speed instruction benefits the development of representations about repeating sequences while forcing participants to be more accurate leads to a faster selection of responses via better stimulus-response associations. In this study, the participants were aware of the repeating sequences; thus, the learning was completely explicit. The studies mentioned above suggest that speed instruction might benefit sequence learning more than accuracy instruction. These studies used relatively simple, deterministic sequences (i.e., sequences with a simple repeating pattern). Therefore, data are still lacking on whether instruction affects probabilistic representations.

Human participants can rapidly extract statistical information from the environment (Frost et al. 2015). But how fragile are these representations? Previous studies have shown that accelerated learning can be advantageous for habit formation (Hardwick et al. 2019) and also affects the sequencing aspect of skill learning (Hoyndorf and Haider 2009; Barnhoorn et al. 2019). However, these studies could not distinguish whether the instructions affect the representations or momentary performance. Instructing participants to be fast or accurate during the learning process, and test their knowledge after the instructed phase would allow us to decipher whether the statistical representations are themselves fragile or only the performance is affected. If instructions do not affect statistical learning, it will underscore the robust nature of picking up non-adjacent statistical regularities (Kóbor et al. 2017).

Here, we aimed to test whether speed or accuracy instructions affect the acquisition of complex statistical regularities using an implicit probabilistic sequence learning task. We go beyond previous investigations by at least 2 aspects: First, by studying complex probabilistic sequences with non-adjacent second-order dependencies (Remillard 2008). This feature means that to predict the nth element of the sequence, we need to know the n-2th element instead of n-1th. This structure creates an abstract sequence representation, and its acquisition will be based on statistical regularities (Nemeth et al. 2013), which are also fundamental in complex cognitive skills such as human language (Christiansen and Chater 2015).

The second novel contribution of our study is that we also test the implicit sequence knowledge of our participants after the (instructed) training phase. Our learning task was completed in 2 different phases. In the first phase, we instructed the participants to focus either on accuracy or speed while performing the task (different instruction phase, accuracy vs. speed group). After the training phase, we tested both groups of participants with the same instruction (i.e., focusing both on accuracy and speed, same instruction phase). By doing so, we aimed to differentiate between the effects of instructions on training performance and acquired knowledge. Our questions were 1) whether the speed/accuracy instruction affects the learning of probabilistic statistical regularities, and if yes, 2) do they affect the training performance (different instruction phase) and the retrieval of knowledge (same instruction phase) equally?

## Materials and Methods

### Participants

Sixty-six healthy young adults took part in the study. Five of them were excluded from the experiment because they conceivably misunderstood the instructions. Their performance was more than 2 standard deviations above or below the mean of their group in more than 50% of the epochs (units of analysis), which was not observable during the practice session. Therefore, 61 participants remained in the final sample (40 females), which is sufficient to detect group differences in statistical learning (see power analysis in the “Justification for sample size” section of the Supplementary Materials). Another 4 participants were excluded from the analysis of the inclusion/exclusion task for not following instructions (see inclusion/exclusion part of the Results section).

Participants were between 19 and 27 years of age (*M*_age_ =21.18 years, SD_age_ = 2.13 years). All of them were students from Budapest, Hungary (*M*_years of education_ = 14.14 years, SD_years of education_ =  1.64 years). Participants had a normal or corrected-to-normal vision, none of them reported a history of any neurological and/or psychiatric disorders, and none of them was taking any psychoactive medication at the time of the experiment. Handedness was measured using the Edinburgh Handedness Inventory (Oldfield 1971). The laterality quotient (LQ) of the sample varied between −84.62 and 100 (−100 indicates complete left-handedness, 100 indicates complete right-handedness, *M*_LQ_ = 62.25, SD_LQ_ = 53.73). They performed in the normal range on the counting span task (*M*_Counting Span_ = 3.66, SD_Counting Span_ = 0.81) All participants gave written informed consent before enrollment and received course credit for participating. They were randomly assigned to the accuracy group (*n* = 31) or speed group (*n* = 30).

No group differences were observed in terms of age, years of education, handedness, and neuropsychological performance (see Table 1). Males and females were equally represented in the sample (accuracy group: 11 males, speed group: 10 males, χ^2^ (1, *N* = 61) = 0.03, *P* = 0.86). The study was approved by the Research Ethics Committee of the Eötvös Loránd University, Budapest, Hungary, and it was conducted in accordance with the Declaration of Helsinki.

**Table 1:** Comparison of the 2 groups on age, years of education, handedness, and neuropsychological performance

	Accuracy group *M*(SD)	Speed group *M*(SD)	*t*-test
Age (years)	21.29 (2.28)	21.07 (2.00)	*t*(59) = −0.41, *P* = 0.69, BF_01_ = 4.82
Education (years)	14.31 (1.60)	13.97 (1.71)	*t*(59) = −0.80, *P* = 0.43, BF_01_ = 3.87
Handedness (LQ)	54.88 (55.00)	69.86 (52.20)	*t*(59) = 1.09, *P* = 0.28, BF_01_ = 3.02
Counting span score	3.69 (0.75)	3.64 (0.88)	*t*(59) = 0.21, *P* = 0.83, BF_01_ = 5.08

### Alternating Serial Reaction Time Task

**Figure 1:**
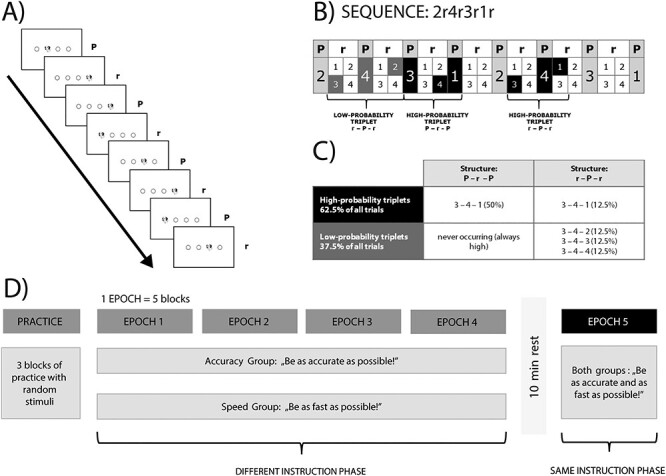
Task and design of the experiment. (*A*) Stimulus presentation in the ASRT task. A dog’s head appeared in one of the 4 positions. Stimuli appeared in either a pattern (P) or a random (r) position, creating an 8-item long alternating sequence structure. (*B*) High- and low-probability triplets. Due to the alternating sequence structure, some runs of consecutive stimuli (called triplets) occurred with a higher probability than others. Every trial was defined as the third element of a high- or a low-probability triplet, based on the 2 preceding trials. High-probability triplets can be formed by 2 patterns and 1 random element, but also by 2 random and 1 pattern element. (*C*) Proportion of high- and low-probability triplets. High-probability triplets occurred in 62.5% of all trials (of which 50% came from pattern elements, i.e., from P-r-P structure, and 12.5% came from random elements, that is, from the r-P-r structure, by chance). Low-probability triplets occurred in the remaining 37.5% of all trials (of which each individual low-probability triplet occurred with a 12.5% probability by chance, originating only from the r-P-r structure). (*D*) Design of the study. In the different instruction phase, different instructions were given to the 2 groups. After 4 epochs (each containing 5 blocks) of the ASRT task, and a 10 min long rest period, the instruction changed. In the fifth epoch (containing 5 blocks of stimuli), the same instruction was given to all of the participants (same instruction phase).

In this study, we used the implicit version of the alternating serial reaction time (ASRT) task (Howard and Howard 1997; Nemeth, Janacsek, Londe, et al. 2010). In the ASRT task, 4 empty circles were presented horizontally in front of a white background in the middle of a computer screen. A target stimulus (drawing of a dog’s head) was presented sequentially in one of the 4 empty circles (Fig. 1*A*). The stimuli were 300 pixels each. The monitor resolution was 1280 × 1024 pixels, and the viewing distance from the monitor was approximately 60 cm. A keyboard with 4 heightened keys (Z, C, B, and M on a QWERTY keyboard) was used as a response device, each of the 4 keys corresponding to the circles in a horizontal arrangement. Participants were asked to respond with their middle and index fingers of both hands by pressing the button corresponding to the target position. At the beginning of each block of the ASRT task, the 4 empty circles appeared horizontally on the screen for 200 ms, and then, the first target stimulus occurred, and it remained on the screen until the first correct response. The next stimulus appeared after a 120 ms response-to-stimulus interval.

The serial order of the 4 possible positions (coded as 1, 2, 3, and 4 in a horizontal arrangement, with 1 as the leftmost and 4 as the rightmost position) in which target stimuli could appear was determined by an eight-element probabilistic sequence. In this sequence, every second element appeared in the same order. In contrast, the other elements’ positions were randomly chosen out of the 4 possible locations (e.g., 2r4r3r1r, where r indicates a truly random position). Therefore, some combinations of 3 consecutive trials (triplets) occurred with a higher probability than others. For example, 2X**4**, 4X**3**, 3X**1**, and 1X**2** (where “X” indicates any possible middle element of the triplet) would often occur because the third element (bold numbers) could be derived from the sequence (or occasionally could be a random element as well). In contrast, 1X**3** or 4X**2** would occur with lower probability because the third element could only be random (Fig. 1*B*). Therefore, the third element of a high-probability triplet is more predictable from the first event when compared to a low-probability triplet.

There were 64 possible triplets in the task (4 stimuli combined for 3 consecutive trials). Sixteen of them were high-probability triplets, each of them occurring in approximately 4% of the trials, 5 times more often than the low-probability triplets. Overall, high-probability triplets occur with approximately 62.5% probability during the task, while low-probability triplets only occur with a probability of 37.5% (Fig. 1*C*).

As participants practice the ASRT task, their responses become faster and more accurate to the high-probability triplets compared to the low-probability triplets, revealing statistical learning throughout the task (Howard and Howard 1997; Song et al. 2007; Kóbor et al. 2017; Unoka et al. 2017). Each block of the ASRT task contained 85 stimuli (5 random trials were presented at the beginning of the block, then the eight-element alternating sequence was repeated 10 times). Each participant performed a randomly selected sequence from the 6 possible original sequences: 2r1r3r4r, 2r1r4r3r, 2r3r4r1r, 2r3r1r4r, 2r4r3r1r, and 2r4r1r3r.

### Inclusion-Exclusion Task

We also administered an inclusion-exclusion task (Destrebecqz and Cleeremans 2001; Destrebecqz et al. 2005; Jiménez et al. 2006; Fu et al. 2010), which is based on the “Process Dissociation Procedure,” a widely used method to disentangle the explicit–implicit processes in memory tasks (Jacoby 1991). In the first part of the task, we asked participants in what order the stimuli (both pattern and random elements) appeared during the task and to type the sequence using the same 4 response buttons they used during the ASRT task (inclusion instruction). After that, they had to generate new sequences that were different from the learned sequence (exclusion condition). Both parts consisted of 4 runs, and each run finished after 24 button presses, which is equal to 3 rounds of the eight-element alternating sequence (Kóbor et al. 2017; Horvath et al. 2018; Kiss et al. 2019).

We assessed performance by the occurrence of high-probability triplets in the sequence of responses. Thus, in the inclusion condition, successful performance is indicated by producing high-probability triplets above chance level. It can be achieved solely by implicit knowledge (however, explicit knowledge can also boost performance, but it is not necessary for the successful completion of the task).

On the contrary, successful performance in the exclusion condition (i.e., generating a new sequence that is different from the learned one) is indicated by the production of high-probability triplets at or under chance level. This is only possible if the participant has conscious (explicit) knowledge about the learned statistical regularities, and they can inhibit the production of high-probability triplets consciously. The generation of the learned statistical regularities above chance level, even in the exclusion task, indicates that the participant relies on their implicit knowledge, as it cannot be controlled consciously.

To test whether the participants gained consciously accessible triplet knowledge, first, we calculated the percentage of the generated high-probability triplets in the inclusion and exclusion conditions separately. Then, we tested whether the occurrence of high-probability triplets differed from the probability of generating them by chance. The chance level was considered 25% because, after 2 consecutive button presses, the chance for the third button press to form a high-probability triplet with the 2 preceding button presses is 1/4 = 25%. We also compared the percentages of the high-probability triplets across conditions (inclusion and exclusion task) and groups (accuracy group and speed group) (for more details about the inclusion-exclusion task, see: Kóbor et al. 2017; Horvath et al. 2018; Kiss et al. 2019).

### Questionnaire

We used a questionnaire to scrutinize whether the participants preferred accuracy or speed in general and whether they were rather accurate or fast in their everyday life. The questionnaire consisted of the following questions: “In an everyday situation, what do you attend more: speed or accuracy (on a scale from 1 to 10, where 1 means that only the accuracy is important and 10 means that only the speed is important)?”, “In an everyday situation, how important is for you to be accurate/fast on a scale from 1 to 10?”, “According to your friends and family, how fast/accurate are you when you need to solve a problem (on a scale from 1 to 10)?”

### Design

First, the participants completed 3 practice blocks of 85 random trials each to familiarize themselves with the task. After that, the participants completed 2 sessions of the ASRT task. In the training session (referred to as different instruction phase), we gave different instructions to the 2 groups. For the accuracy group, the instruction was to try to be as accurate as possible during the task. On the contrary, the instruction for the speed group was to be as quick as possible. Twenty blocks were presented to the participants in the different instruction phase (for analysis, we organized the blocks into 4 epochs by merging 5 consecutive blocks). Participants could rest a bit after each block. A 10 min rest period was inserted before the second ASRT session. During this period, participants were not involved in any demanding cognitive activity. The second session of ASRT (referred to as the same instruction phase) contained 5 blocks (one epoch). This time, both the accuracy and speed group were instructed to respond to the target stimulus as quickly and as accurately as possible (Fig. 1*D*). After the ASRT task, the inclusion-exclusion task was administered.

### Statistical Analysis

We defined each trial as the third element of a high- or low-probability triplet. Trills (e.g., 1-2-1) and repetitions (e.g., 1-1-1) were eliminated from the analysis because participants tended to show preexisting response tendencies to these types of triplets (Howard et al. 2004; Unoka et al. 2017; Janacsek et al. 2018; Takács et al. 2018). The first 5 button presses were random; thus, only the eighth button press could be evaluated as the last element of a valid triplet. Therefore, the first 7 trials were excluded from the analysis. Blocks were collapsed into 4 epochs in the different instruction phase (Epoch 1–4), and one epoch in the same instruction phase (Epoch 5) to facilitate data processing and to reduce intraindividual variability. We calculated the median reaction times (RTs) separately for high- and low-probability triplets for each participant and each epoch. Only correct responses were considered for the RT analysis (we also performed the analyses with the incorrect trials included, see “Analyses including the incorrect trial*s*” in the Supplementary Materials). To ensure that our results on the learning measures were not due to the differences in the average RTs and accuracies, we repeated the analyses with standardized scores (for details, see “Standardized learning scores” section in the Supplementary Materials).

We used mixed-design ANOVAs to compare the learning performance between the 2 groups in the different and same instruction phase. ANOVAs with the within-subject factor of triplet (high- vs. low-probability triplets) and the between-subjects factor of group (accuracy group vs. speed group) were run (and also with the epoch factor for the analysis of the different instruction phase). In all ANOVAs, the Greenhouse–Geisser epsilon (ε) correction was used if necessary. Corrected df values and corrected *P* values are reported (if applicable) along with partial eta-squared (*η_p_*^2^) as the measure of effect size. We used the least significant difference tests for pairwise comparisons. Significant interactions involving the triplet factor were further analyzed using follow-up ANOVAs on the difference scores by the Triplet factor (high-probability triplets vs. low-probability triplets).

To further support the results of our comparisons, we ran Bayesian *t*-tests with a standard Cauchy prior distribution (*r* = 1) (Rouder et al. 2009). Here, we report BF_01_ values: greater values support the null hypothesis over the alternative hypothesis. BF_01_ values between 1 and 3 indicate anecdotal evidence for H_0_, while values between 3 and 10 indicate substantial evidence for H_0_. Values between 1 and 0.33 indicate anecdotal evidence for H_1_, values between 0.33 and 0.1 indicate substantial evidence for H_1_. BF_01_ values around one do not support either H_0_ or H_1_ (Wagenmakers et al. 2011).

To obtain a robust indication of which factors determine performance, we also performed Bayesian repeated-measures ANOVAs on the learning scores (the difference between the 2 levels of triplet factor, i.e., learning scores) (Zavecz et al. 2020). We decided to run the ANOVAs on the learning scores because our primary interest was to quantify the contribution of each interaction to statistical learning rather than to general RTs. Here, we present Bayesian Model Averaging and report the inverted BF inclusion values (1/BF_inclusion_). These values indicate the amount of evidence for the exclusion of the given factor from our model. Thus, values below 1 support the inclusion and values above 1 the exclusion of the given factor. Full model comparisons are included in the Supplementary Materials (see “Model comparisons of statistical learnin*g*” section in the Supplementary Materials). Cauchy prior distribution was used for the ANOVA with a fixed-effects scale factor of *r* = 0.5, and a random-effects scale factor of *r* = 1 (JASP Team 2020).

To test whether participants developed conscious knowledge about the learned statistical regularities, we compared the percentage of the generated high-probability triplets in the inclusion-exclusion task to chance level (25%) separately for the 2 groups with one-sample *t*-tests. We compared the percentage of high-probability triplets with a mixed-design ANOVA to reveal whether the level of explicitness differs between groups and conditions.

Additionally, we correlated the average RTs and accuracy scores with the rates of the different items of the questionnaire to check whether the subjective preferences of the participant are related to the ability to follow the instructions.

## Results

### Did the Two Groups Perform Equally Before Learning?

To ensure the lack of substantial preexisting differences between groups in terms of speed or accuracy, we compared the median RTs (only for correct responses) and the accuracy of the 2 groups in the practice session (random stimuli). We did not find differences between groups either in RTs, *t*(59) = 0.48, *P* = 0.64, BF_01_ = 4.67, or in accuracy measures, *t*(59) = 1.08, *P* = 0.28, BF_01_ = 3.04. Therefore, we assumed that there were no preexisting differences between groups regarding their speed or accuracy.

### General Speed Changes and Statistical Learning in RT Measures in the Different Instruction Phase

We investigated how 1) general RTs changed, and 2) whether statistical learning differed between groups during the different instruction phase. RTs were analyzed with a mixed-design ANOVA with the within-subject factors of triplet (high- vs. low-probability triplets) and epoch (Epoch 1–4), and the between-subjects factor of group (accuracy group vs. speed group). Please note that main effects and interaction excluding the triplet factor could reveal changes in the average speed/accuracy during the task, independent of the acquisition of statistical regularities, and the main effects and interaction including the triplet factor could unveil differences in statistical learning.

We also compared the learning process with standardized learning scores (see Materials and Methods section). To this end, a mixed-design ANOVA was performed on the standardized RT learning scores with epoch (Epoch 1–4) as a within-subject factor and group (accuracy group vs. speed group) as a between-subjects factor.

#### Did the Instruction Affect General RTs in the Different Instruction Phase?

**Figure 2:**
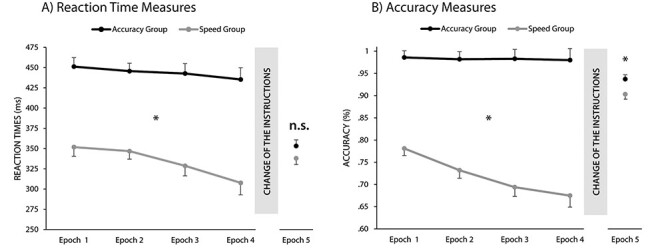
Effects of instruction on (*A*) average RTs and (*B*) accuracies. The horizontal axis indicates the 5 epochs of the task and the vertical axis the RTs in milliseconds/accuracies in percentage. The error bars represent the standard error of the mean (SEM). Average RTs were significantly shorter and accuracies lower for the speed group from the first epoch, indicating that the participants followed the instructions. After the change of the instructions (Epoch 5)—although the average scores of the 2 groups approached each other—the difference persisted for accuracies; however, the difference disappeared for the average RTs. ^*^ = *P* < 0.05, n.s. = *P* > 0.05.

The main effect of group was significant, *F*(1, 59) = 51.86, *P* < 0.001, *η_p_*^2^ = 0.47, indicating faster overall RTs in the speed group, and the Bayesian comparison of means also favored the difference, BF_01_ < 0.001; thus, the instruction did modify the average speed of the participants. A main effect of epoch was found, *F*(1.97, 116.33) = 7.46, *P* = 0.001, *η_p_*^2^ = 0.11, indicating a change in average RTs during the task: significantly faster RTs were observed between Epoch 2 and Epoch 3 (*P* = 0.008) as well as between Epoch 3 and Epoch 4 (*P* = 0.049). The epoch × group interaction was non-significant, *F*(1.97, 116.33) = 2.30, *P* = 0.10, *η_p_*^2^ = 0.04 (Fig. 2).

#### Did Statistical Learning Measured by RTs Differ Between Groups in the Different Instruction Phase?

**Figure 3:**
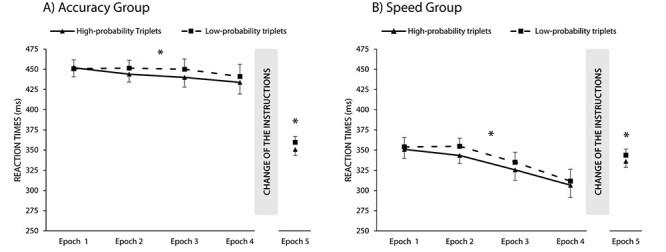
Learning in RT measures in the (*A*) accuracy group and (*B*) speed group. The horizontal axis shows the 5 epochs of the task and the vertical axis the RTs. The solid line represents the RTs for the high-probability triplets, while the dashed line indicates the RTs for the low-probability triplets. The error bars represent the SEM. Please note that the gap between the 2 lines indicates the learning of statistical regularities. The RTs for high-probability triplets were smaller for both groups and phases. The difference between the 2 trial types remained after the change of the instructions. A similar level of learning was measured in both groups and phases. ^*^P < 0.05.

The main effect of triplet was significant, *F*(1, 59) = 49.41, *P* < 0.001, *η_p_*^2^ = 0.46: faster RTs were found for high-probability triplets compared to low-probability triplets (BF_01_ < 0.001), revealing significant implicit statistical learning. Importantly, the triplet × group interaction was non-significant, *F*(1, 59) = 0.48, *P* = 0.49, *η_p_*^2^ = 0.01: the degree of learning did not differ between the 2 groups over the course of the learning. The Bayesian comparison of mean differences also supported the lack of difference, BF_01_ = 4.17 (Fig. 3). The triplet × epoch interaction was significant, *F*(3, 177) = 5.66, *P* = 0.001, *η_p_*^2^ = 0.09: In the first epoch, independently from groups, no difference was detected between high- and low-probability triplets (*P* = 0.54), and learning (faster RTs for high- than for low-probability triplets) emerged from the second epoch (each *P* < 0.007). Follow-up analysis on the difference between high- and low-probability triplets (learning scores) revealed an increase in learning scores between Epoch 1 and Epoch 2 (*P* < 0.001), but not between Epoch 2 and Epoch 3 (*P* = 0.90) or Epoch 3 and Epoch 4 (*P* = 0.17). The interaction between the triplet, epoch, and group factors was non-significant, *F*(3, 177) = 0.90, *P* = 0.43, *η_p_*^2^ = 0.02, revealing no difference in the time course of statistical learning between groups. The analysis with the standardized learning scores in the RT measures revealed similar results (see Supplementary Materials for details).

#### Bayesian Model Averaging in the Different Instruction Phase in RT Measures

We conducted a Bayesian repeated-measures ANOVA to quantify the contribution of the different factors to statistical learning (to the difference between the 2 levels of the Triplet factor, that is, low-probability triplets minus high-probability triplets). The ANOVA was performed on the learning scores as the dependent variable, with the within-subject factor of epoch (Epoch 1–4) and the between-subject factor of group (accuracy group vs. speed group). Please note that, because this ANOVA is conducted on learning scores, here the epoch factor corresponds to the triplet × epoch interaction, the group factor to the triplet × group interaction, and the epoch × group interaction to the three-way interaction of the frequentist ANOVA. The Bayesian ANOVA supported the inclusion of the epoch factor, and the exclusion of the group factor and the epoch × group interaction (Table 2). This result suggests that although the learning scores changed throughout the task, this change was independent of the instructions, and the overall statistical knowledge was not different between the 2 groups (see detailed model comparisons in Supplementary Table 7).

**Table 2:** Analysis of effects for the RT learning scores

Effects	*P*(incl)	*P*(incl|data)	BF_exclusion_
Epoch	0.60	0.98	0.04
Group	0.60	0.19	6.49
Epoch × Group	0.20	0.02	11.13

Notes: The column ``Effects'' lists the main effects and interactions. The *P*(incl) column denotes the prior, and the *P*(incl|data) the posterior inclusion probability. The BF_exclusion_ column indicates the change from prior to posterior odds.

### General Accuracy Changes and Statistical Learning in Accuracy Measures in the Different Instruction Phase

Next, we repeated the above analyses on accuracy measures to see how 1) general accuracy changed, and 2) whether statistical learning differed between groups during the different instruction phase. We ran a mixed-design ANOVA with the within-subject factors of triplet (high- vs. low-probability triplets) and epoch (Epoch 1–4), and the between-subject factor of group (accuracy group vs. speed group). Please note again that the main effects and interaction excluding the triplet factor could reveal information about the average speed/accuracy during the task, independent of statistical learning, and main effects and interaction including the triplet factor could unveil potential differences in terms of statistical learning.

#### Did the Instruction Affect General Accuracies in the Different Instruction Phase?

The main effect of group was significant, *F*(1, 59) = 117.40, *P* < 0.001, *η_p_*^2^ = 0.67, signaling higher average accuracy in the accuracy group; thus, the instructions did influence the accuracy of the participants. The Bayesian comparison of means also supported the difference (BF_01_ < 0.001). The ANOVA revealed a main effect of epoch, *F*(1.81, 107 = 8.19, *P* = 0.001, *η_p_*^2^ = 0.13, revealing a significant decrease in accuracies between Epoch 1 and Epoch 2 (*P* = 0.02) and between Epoch 2 and Epoch 3 (*P* = 0.002). The epoch × group interaction was significant, *F*(1.84, 107) = 7.08, *P* = 0.002, *η_p_*^2^ = 0.11, indicating that accuracy decreased over the epochs in the speed group (each *P* < 0.005, except between Epoch 3 and Epoch 4, *P* = 0.36), and it remained similarly high in all epochs in the accuracy group (each *P* > 0.74) (Fig. 2).

#### Did Statistical Learning Measured by Accuracies Differ Between Groups in the Different Instruction Phase?

**Figure 4:**
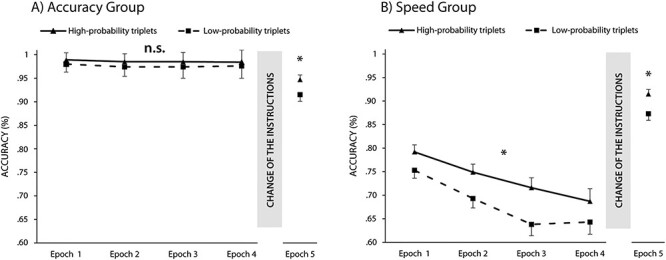
Learning in accuracy measures in the (*A*) accuracy group and (*B*) speed group. The horizontal axis shows the 5 epochs of the task and the vertical axis the RTs. The solid line represents the RTs for the high-probability triplets, while the dashed line indicates the RTs for the low-probability triplets. The error bars represent the SEM. Please note that the learning of statistical regularities is measured by the gap between the 2 lines. The accuracies for high-probability triplets were smaller in the speed group, but not in the accuracy group. However, learning was measurable in both groups after the change of the instructions. ^*^ = *P* < 0.05, n. s. = *P* > 0.05.

The main effect of triplet was significant, *F*(1, 59) = 93.88, *P* < 0.001*, η_p_*^2^ = 0.61: participants responded more accurately to high-probability triplets compared to low-probability triplets, revealing prominent implicit statistical learning also in accuracy measures. The Bayesian comparison also supported the difference, BF_01_ < 0.001. Contrary to the RT results, the ANOVA revealed a significant interaction between the triplet and group factors, *F*(1, 59) = 45.25, *P* < 0.001, *η_p_*^2^ = 0.43. The speed group responded more accurately to high-probability triplets compared to the low-probability triplets; the accuracy group exhibited similarly accurate responses to the 2 types of triplets (BF_01_ < 0.001) (Fig. 4). The triplet × epoch interaction was significant, *F*(3, 177) = 3.69, *P* = 0.01, *η_p_*^2^ = 0.06; thus, the degree of statistical learning changed over the course of learning. Follow-up analysis of the difference between high- and low-probability triplets (learning scores) revealed a decrease in statistical knowledge between Epoch 3 and Epoch 4 (*P* = 0.01), but not between Epoch 1 and Epoch 2 (*P* = 0.19) or Epoch 2 and Epoch 3 (*P* = 0.13). The triplet × epoch × group interaction was also significant, *F*(2.95, 174.28) = 2.99, *P* = 0.03, *η_p_*^2^ = 0.05, suggesting different dynamics of implicit statistical learning for the 2 groups. The follow-up analysis on the difference between high- and low-probability triplets (learning scores) revealed that in the accuracy group, no change was observed between consecutive epochs (each *P* > 0.74). On the contrary, in the speed group, an increase was observed between Epoch 2 and Epoch 3 (*P* = 0.04) and a decrease between Epoch 3 and Epoch 4 (*P* = 0.001). The analysis with the standardized learning scores in accuracy measures revealed similar results (see Supplementary Materials for details).

#### Bayesian Model Averaging in the Different Instruction Phase in Accuracy Measures

We ran a Bayesian repeated-measures ANOVA on the accuracy learning scores with the same factors as for the RT analysis. The Bayesian ANOVA indicates that, averaged across all models, the models including the group factor, the epoch factor, and the interaction are more likely. However, the latter 2 improve the model to a much smaller extent compared to the Group factor. This result underscores that the instructions affected statistical learning in accuracy measures, and the dynamic of the learning trajectory is different between the 2 groups (see detailed model comparisons in Supplementary Table 8) (Table 3).

**Table 3:** Analysis of effects for the accuracy learning scores

Effects	*P*(incl)	*P*(incl|data)	BF_exclusion_
Epoch	0.60	0.80	0.38
Group	0.60	1.00	9.50e − 7
Epoch × Group	0.20	0.47	0.29

Notes: The column ``Effects'' indicates the main effects and interactions. The *P*(incl) column denotes the prior, and the *P*(incl|data) the posterior inclusion probability. The BF_exclusion_ column indicates the change from prior from posterior odds.

### Did the Acquired Knowledge Differ Between Groups in the Same Instruction Phase?

First, we calculated the median RTs separately for the high- and low-probability triplets in the same instruction phase. We analyzed RTs of Epoch 5 with a mixed-design ANOVA with the within-subject factor of triplet (high-probability triplets vs. low-probability triplets) and the between-subjects factor of group (accuracy group vs. speed group).

**Figure 5:**
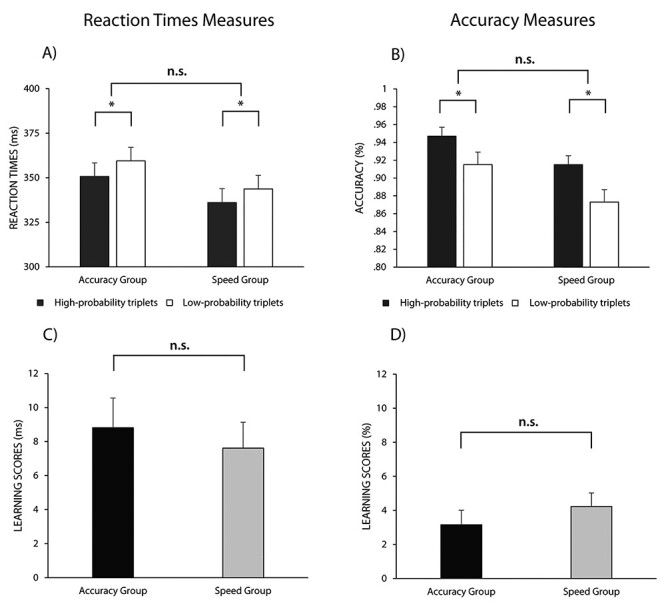
Comparison of the high- and low-probability triplets (*A* and *B*), and the learning scores in the same instruction phase. The vertical axis indicates the RTs (*A*), accuracy (*B*), or the learning scores (the difference between high- and low-probability triplets, C and D). The horizontal axis represents the 2 groups. The error bars denote the SEM. Although statistical knowledge was detected in both groups, no significant difference was found in the learning scores, and the lack of difference was confirmed by Bayesian analysis. ^*^ = *P* < 0.05, n.s. = *P* > 0.05.

A significant main effect of triplet was found, *F*(1, 59) = 50.50, *P* < 0.001, *η_p_*^2^ = 0.46, indicating the emergence of statistical knowledge, as RTs for high-probability triplets were smaller than RTs for low-probability triplets (BF_01_ < 0.001). The main effect of group did not reach significance, *F*(1, 59) = 2.03, *P* = 0.16, *η_p_*^2^ = 0.03, indicating that after the change of the instructions, the average RT difference between the 2 groups disappeared; however, the Bayesian comparison revealed only anecdotal evidence for the lack of difference, BF_01_ = 2.08 (Fig. 2). Importantly, the triplet × group interaction did not reach significance, *F*(1, 59) = 0.27, *P* = 0.60, *η_p_*^2^ = 0.01. It indicates that, irrespective of the instruction during training, the 2 groups showed the same level of statistical knowledge in the same instruction phase (Fig. 5). Moreover, the Bayesian comparison of statistical learning (the difference between high- and low-probability triplets) between groups also favored the lack of difference, BF_01_ = 4.58. The analysis with the standardized learning scores in the RT measures revealed similar results (see Supplementary Materials for details).

Next, we repeated the above analysis on the accuracy scores. The triplet × group ANOVA revealed a significant main effect of triplet, *F*(1, 59) = 39.96, *P* < 0.001, *η_p_*^2^ = 0.40, indicating statistical knowledge in accuracy as well: more accurate responses for high-probability triplets compared to the low-probability triplets (BF_01_ < 0.001). The main effect of group was also significant, *F*(1, 59) = 5.08, *P* = 0.03, *η_p_*^2^ = 0.08, indicating that the overall difference in accuracy persisted after the change of the instructions; however, according to the Bayesian *t*-test, the difference was only anecdotal (BF_01_ = 0.55). Importantly, the triplet × group interaction did not reach significance, *F*(1, 59) = 0.85, *P* = 0.36, *η_p_*^2^ = 0.01, indicating a similar level of statistical knowledge after the change of the instructions (Fig. 5). The Bayesian comparison of statistical learning between groups also supported the lack of difference, BF_01_ = 3.53. The analysis with the standardized learning scores in accuracy measures revealed comparable results (see Supplementary Materials for details).

### Did the Participants Develop Conscious Knowledge about the Statistical Regularities, and was it Different Between Groups?

The inclusion-exclusion task was administered to reveal whether the acquired statistical knowledge remained implicit or became explicitly accessible for the participants. We compared the percentage of the generated high-probability triplets to the chance level separately for the 2 groups (see Materials and Methods section for details).

In the accuracy group, 2 participants were excluded from this analysis because they did not follow the instructions. Participants in the accuracy group generated 32.33% (0.15% SEM) high-probability triplets in the Inclusion condition, which is significantly higher than chance level, *t*(28) = 4.82, *P* < 0.001, BF_01_ = 0.002. In the exclusion condition, they generated 29.81% (0.12% SEM) high-probability triplets, which is significantly above chance level, *t*(28) = 4.04, *P* < 0.001, BF_01_ = 0.01, indicating that they could not consciously inhibit the emergence of this knowledge. These results show that in the accuracy group, knowledge about the statistical regularities remained implicit.

In the speed group, 2 participants were excluded because they did not follow the instructions. Participants in the speed group generated 30.34% (0.15% SEM) high-probability triplets in the inclusion condition, which is significantly above the chance level, *t*(27) = 3.58, *P* = 0.001, BF_01_ = 0.04. They also generated more high-probability triplets than expected by chance in the exclusion condition, 29.25% (0.21% SEM), *t*(27) = 2.07, *P* = 0.048, BF_01_ = 0.99; thus, knowledge about the statistical regularities remained implicit in the speed group.

Furthermore, we compared the differences between groups and tasks with a 2 (condition: inclusion vs. exclusion) × 2 (group: accuracy group vs. speed group) ANOVA. The main effect of condition was not significant, *F*(1, 55) = 1.66, *P* = 0.20, *η_p_*^2^ = 0.03, indicating that participants did not perform better in either condition, which was confirmed by a Bayesian *t*-test, BF_01_ = 4.21. Thus, the triplet knowledge of the participants remained implicit. The group main effect did not reach significance, *F*(1, 55) = 0.53, *P* = 0.47, *η_p_^2^* = 0.01, indicating that the 2 groups performed equally on the 2 tasks, confirmed also by the Bayesian *t*-test, BF_01_ = 3.96. The interaction of the condition and group factors was not significant, *F*(1, 55) = 0.26, *P* = 0.61, *η_p_*^2^ = 0.01, revealing that the lack of difference between groups was not influenced by the type of task (BF_01_ = 4.47). To sum up, the 2 groups performed similarly on the task.

### Did the Preexisting Preferences of the Participants Affect Their Performance on the Task?

We used a questionnaire to check whether the subjective preferences on being fast or accurate in real life were related to the ability to follow instructions (see Materials and Methods section for the questions). We correlated the questionnaire scores with the average RTs and accuracy of the participants separately for the 2 groups. We did not find any significant correlations between the average scores and subjective ratings either in the accuracy group or in the speed group (each *P* > 0.09). This result indicates that the preference for accuracy or speed, and whether the participants are rather fast or accurate in real life did not play a role in the ability to follow the instructions.

## Discussion

Here, we aimed to unveil whether speed/accuracy instructions can influence an essential component of skill learning, namely the acquisition of probabilistic statistical regularities. To this end, we instructed 2 groups of participants to be either fast or accurate during the training on an implicit probabilistic sequence learning task (different instruction phase). In the testing phase, we assessed the acquired knowledge of probabilistic regularities, and this time, all participants were instructed to be both fast and accurate (same instruction phase). As predicted, the instructions greatly affected the average speed and accuracy of the participants: the speed instructions resulted in faster RTs and a higher number of errors, while the accuracy instructions caused slower average RTs and an almost errorless performance. Despite these differences during training, the statistical learning scores based on RTs were similar between groups. However, statistical learning was not detectable with accuracy instructions. Thus, measured by RTs, the instructions did not affect the acquisition of implicit probabilistic regularities during training. Moreover, no difference between the groups was found in the testing phase. This lack of difference suggests that instructions did not affect either the performance during training or the acquired statistical knowledge. Similar results were obtained when we controlled for the differences in average speed between groups. Moreover, Bayesian statistical methods also supported the lack of difference between groups in terms of acquired knowledge.

Our main result is that, irrespective of the strategy used during the training, we detected a similar level of acquired statistical knowledge. This finding has several implications. From a narrower, learning perspective, it suggests that our ability to extract the relevant pieces of statistical information from the environment is so robust that instructions cannot influence it. This conclusion is in accordance with the findings that statistical knowledge persists and remains resistant to interference even after 1 year (Kóbor et al. 2017), is intact in dual-task conditions (Vékony et al. 2019) or in certain disorders characterized by cognitive dysfunctions, such as obstructive sleep apnea (Nemeth et al. 2012; Csabi et al. 2014), sleep-disordered breathing (Csábi et al. 2013, 2016), autism (Nemeth, Janacsek, Balogh, et al. 2010), borderline personality disorder (Unoka et al. 2017) or alcohol dependency (Virag et al. 2015). Deterministic learning tasks test patterns that occur with a 100% probability over time, while the alteration of the random and pattern elements in the ASRT task creates a noisy, uncertain environment, which is similar to the natural environments of learning in everyday life (Fiser et al. 2010). Our results showed that using complex probabilistic regularities, a similar level of statistical knowledge emerges throughout learning, even when learning occurs under different circumstances and with different strategies.

Another compelling result of our study is that participants in the accuracy instruction condition acquired stable statistical knowledge despite the minimization of motor (response) errors during training. The extent of this statistical knowledge was comparable with the knowledge acquired with the speed instruction. This result is especially interesting in light of the theory claiming that the brain is a Bayesian inference machine (Friston 2010) because our results contradict to the findings that committing errors facilitates learning (Bubic et al. 2010). Our brain learns associations between events through continuous adjustments of the estimated probability distribution, that is, the prior. After a prediction error, the prior should be updated in accordance with new information about the probabilistic structure (Friston 2010). Based on these theories, we would expect a low number of errors to impair the learning process; however, this was not the case in our study. This finding raises the possibility that the motor aspect of prediction errors is not crucial in all circumstances for updating the priors during probabilistic sequence learning. This claim is also supported by other studies reporting statistical and sequence learning without overt errors (Fiser and Aslin 2001; Aslin 2017). However, it is also possible that a similar amount of prediction errors might be detected with other methods, for example, by investigating eye movements (Wills et al. 2007; Le Pelley et al. 2011). The exploration of the role of errors in implicit statistical learning deserves future investigation using eye-tracking and electrophysiological methods.

Measured by RTs, a similar level of statistical learning was found under the speed and accuracy instruction conditions in the training phase. This finding is in contrast with the results of Hoyndorf and Haider (2009), as they reported impaired implicit learning performance with an accuracy strategy. In their study, participants performed a regular and a random task set during a number reduction task. They found that only the participants focusing on speed had increased speed for the regular task set. The authors claimed that the increased monitoring due to the accuracy instruction might have impeded the performance, similarly to the results of skill acquisition studies (Beilock et al. 2004, 2008). However, in the same study, Hoyndorf and Haider (2009) found a preference for the regular task set also in the accuracy group, which they interpreted as the focus on accuracy affects only the expression of implicitly acquired knowledge rather than learning processes per se. This conclusion is in accordance with our results, as we found a similar level of statistical knowledge when we equally emphasized the importance of speed and accuracy after the initial learning. The difference in the training phase might be due to the more complex, probabilistic sequence representations used in our study. They may be more resistant to instructions than deterministic patterns. Similarly, Barnhoorn et al. (2019), who have also found the speed instruction to benefit the development of sequence representations, used simple repeating sequences. Moreover, this study investigated explicit sequence learning processes, while our participants were unaware of their accumulated statistical knowledge. A possible explanation for the difference between the effect of implicit and explicit learning conditions could be that the increased speed covers up the explicitness of the task. As a consequence, the task becomes more implicit, the top-down control reduces, and the learning becomes better. In our study, the learning was entirely implicit; therefore, the speeding up could not improve the level of implicitness. Thus, the learning was similar under speed and accuracy instructions. Future investigations are needed to determine the extent to which the implicit or probabilistic nature of the task affects the lack of speed benefit during training.

Although we found a similar level of the acquired statistical knowledge in accuracy measures, a difference was revealed in the training performance: only the speed instruction resulted in measurable statistical learning. Accuracy is a measure that can reach a maximum of 100%; that is, the task can be performed without errors. Our results suggest that the accuracy instruction caused a ceiling effect. Participants completed the task nearly without error, which did not allow us to measure statistical learning in accuracy measures (i.e., to find a significant difference between responses to high- vs. low-probability triplets). However, learning did occur, evidenced by the results of the testing phase. These findings call for a more careful approach when we evaluate the learning phase in terms of accuracy measures: focusing on being accurate can distort the learning scores of interest so much that, in some instances, we cannot reveal the knowledge that exists.

From a broader cognitive neuroscience perspective, it is essential to highlight the relationship between learning and performance in our study. Most studies in the field of cognitive neuroscience measure learning in a single context, and draw conclusions about brain-behavior relationships based on either “long-term learning” (the relatively permanent changes in knowledge, i.e., competence) or “momentary performance” (the temporary fluctuation in behavior) (e.g., Thomas et al. 2004; Turk-Browne et al. 2010; Rose, Haider, Salari, and Buchel 2011; Heideman, van Ede, and Nobre 2018). However, it was shown that these 2 factors could be separated from each other. For example, learning and performance can differ due to fatigue, different types of practice, latent learning, or overlearning of the practiced skill (Soderstrom and Bjork 2015). Our study also revealed that skill learning competence could differ from the momentary performance due to different instructions, at least when accuracy is used as an indicator. This result draws attention to the problem of using only one session to evaluate learning. For example, if the fatigue or boredom of the participants are different when they concentrate on being fast or accurate, then it can influence the conclusions we draw from our results. However, when the learning score (difference score) is based on RTs, this contingency appears smaller, at least when investigating implicit probabilistic sequence learning. Future studies should reveal to what extent this phenomenon is generalizable to other types of learning, such as to more explicit or non-statistical learning tasks. Non-learning tasks should also be tested, as general speed-up and changes in accuracy can be seen over the course of various cognitive tasks requiring fast decision-making. Based on our results, we recommend taking into consideration the possible differences between the measured competence and performance when designing learning studies.

We manipulated the general speed and accuracy of the participants by giving explicit instructions to focus either on speed or accuracy, as previous non-learning cognitive tasks also did (e.g., Osman et al. 2000; Christensen et al. 2001; Ullsperger et al. 2004; Aasen and Brunner, 2016). However, it might be questionable if our results genuinely reflect the effect of instructions on learning. One can argue that the instructions in our study were not strong enough to manipulate the learning strategy and the learning processes because previous studies used more pronounced instructions and feedback to modify the strategy of the participants (Hoyndorf and Haider 2009; Barnhoorn et al. 2019). This possibility seems unlikely because, based on our results, the average speed and accuracy were affected by the instructions. Group differences also emerged in “general skill learning” as 1) participants who focused on their speed showed increasingly faster responses, and 2) participants who focused on their accuracy sustained a high level of accuracy during the learning phase compared to the other group. In contrast to these findings, the acquisition of statistical regularities was not affected by the instructions. To sum up, we found evidence that speed and accuracy affect general skill learning and statistical learning differently.

One could also argue that verbal instructions given at the beginning of the task might not be sufficient to regulate subjects’ average speed and accuracy because, as time goes on, participants tend to wane in favor of their response tendencies (Heitz 2014). In other words, they will behave according to their preferences for being accurate or fast on a task. In our case, this change in behavior seems unlikely. First, we found no differences in the average RTs and accuracy scores between groups when the participants practiced the task on random sequences (before we gave distinct instructions to the groups), and second, participants did not become less accurate or slower throughout the task. Therefore, the observed effects should be the result of the instructions. Additionally, we measured the participants’ individual preferences on response tendencies using a questionnaire (whether they preferred to be accurate or fast). No correlations were observed between these individual preferences and the average speed and accuracy during the task in either group. These aspects indicate that our results indeed reflect the effect of instructions, and participants did not follow their individually preferred response tendencies during the task.

## Conclusion

Our study investigated the effects of speed and accuracy instructions on an essential component of skill learning, namely, the acquisition of probabilistic regularities. Our main finding is that our ability to pick up statistical regularities in a noisy, uncertain environment is so robust that instructions do not influence it. This result indicates that implicit probabilistic sequence learning is independent of the manipulation of the speed/accuracy trade-off. Another finding of our study is that learning can occur with an almost 100% accuracy level as well. This result suggests that statistical learning is at least partly independent of accuracy level, and statistical knowledge about the environmental regularities can be acquired even if no response (motor) errors occur. Our results also raise the possibility that competence and performance can differ in some instances. Accuracy instructions can mask the accumulating statistical knowledge during learning when measured by accuracy, although knowledge does emerge in these cases as well. Future studies investigating whether this robustness is related to the implicit feature of the task or whether different types of learning are affected equally seem warranted.

## Notes

The authors are grateful to Lucia Nemes, Soma Beres, and Reka Sefcsik for their help in data acquisition. *Conflict of Interest*: None declared.

## Funding

National Brain Research Program (project 2017-1.2.1-NKP-2017-00002); Hungarian Scientific Research Fund (NKFIH-OTKA K 128016, PI: D. N., NKFIH-OTKA PD 124148, PI: K.J.); Janos Bolyai Research Fellowship of the Hungarian Academy of Sciences (to K.J. and A.M.); EFOP-3.6.1-16-2016-00008 (to A.M.); IDEXLYON Fellowship of the University of Lyon as part of the Programme Investissements d'Avenir (ANR-16-IDEX-0005) (to D.N).

## Supplementary Material

Supplementary_Materials_tgaa041
