# Supplementary material for: Speed or Accuracy Instructions During Skill Learning do not Affect the Acquired Knowledge
Source: Cereb Cortex Commun. 2020 Aug 10;1(1):tgaa041. doi: 10.1093/texcom/tgaa041 (PMC8152873; doi:10.1093/texcom/tgaa041)
Supplement: Supplementary_Materials_tgaa041 [file supplementary_materials_tgaa041.docx]

Supplementary Materials

**Speed or accuracy instructions during skill learning do not affect the acquired knowledge**

Teodóra Vékony^a^, Hanna Marossy^b^, Anita Must^c^, László Vécsei^a,d^, Karolina Janacsek^b,e,f*^ & Dezső Németh^b,e,g*^

^a^ Department of Neurology, University of Szeged, Szeged, Semmelweis utca 6, 6725 Szeged, Hungary

^b^ Institute of Psychology, ELTE Eötvös Loránd University, Izabella utca 46, 1064, Budapest, Hungary

^c^ Institute of Psychology, University of Szeged, Egyetem utca 2, 6722 Szeged, Hungary

^d^ MTA-SZTE Neuroscience Research Group, Semmelweis u. 6, H-6725 Szeged, Hungary

^e^ Brain, Memory and Language Research Group, Institute of Cognitive Neuroscience and Psychology, Research Centre for Natural Sciences, Magyar tudósok körútja 2, H–1117 Budapest, Hungary

^f^ Centre for Thinking and Learning, Institute for Lifecourse Development, School of Human Sciences, Faculty of Education, Health and Human Sciences, University of Greenwich, Old Royal Naval College, 150 Dreadnought, Park Row, SE10 9LS, London, United Kingdom

^g^ Lyon Neuroscience Research Center (CRNL), INSERM, CNRS, Université Claude Bernard Lyon 1, Centre Hospitalier Le Vinatier - Bâtiment 462 - Neurocampus 95 boulevard Pinel 69675 Bron, France

^*^ These authors contributed equally to this work.

**Author Note**

Correspondence concerning this article should be addressed to Dezso Nemeth, Lyon Neuroscience Research Center (CRNL), INSERM, CNRS, Université de Lyon, Centre Hospitalier Le Vinatier - Bâtiment 462 - Neurocampus 95 boulevard Pinel 69675 Bron, France. E-mail: dezso.nemeth@univ-lyon1.fr Phone: +33 4 81 10 65 46

# Justification for sample size

We ran a power analysis with G*Power 3.1.9.7 to justify our sample size (Faul et al. 2007). We calculated the required sample size based on the results of previously published ASRT studies with a significant interaction between the within-subject factor of Triplet (high- vs. low-probability) and the between-subjects factor of Group with two independent groups (Nemeth, Janacsek, and Fiser 2013; Nemeth, Janacsek, Király, et al. 2013; Nemeth, Janacsek, Polner, et al. 2013; Virag et al. 2015). The effect sizes ranged from a *η*_p_^2^ effect size of .12 to .35; therefore, we estimated a η_p_^2^ effect size of .21 (mean of the previous effect sizes). With an alpha level of .05 and the desired power level of .90, a total sample size of at least 46 participants is required to detect significant group differences in triplet learning, and 54 participants are required with a power level of .95. Moreover, if we calculate with the lowest expected effect size (*η*_p_^2^ = .12), we would need 60 participants to find a difference with a power of .80, which is the commonly recommended value (Ellis 2010). Therefore, our sample of 61 participants should be sufficient to detect group differences in triplet learning.

**Model comparisons of statistical learning**

| **Supplementary Table 1. Bayesian model comparisons for RT learning scores** | | | | | | | | | | | |
| --- | --- | --- | --- | --- | --- | --- | --- | --- | --- | --- | --- |
| **Models** | | **P(M)** | | **P(M\|data)** | | **BF _M_** | | **BF _01_** | | **error %** | |
| Null model (incl. subject) |  | 0.20 |  | 0.02 |  | 0.08 |  | 1.00 |  |  |  |
| Epoch |  | 0.20 |  | 0.79 |  | 15.38 |  | 0.02 |  | 0.56 |  |
| Epoch + Group |  | 0.20 |  | 0.16 |  | 0.77 |  | 0.12 |  | 1.17 |  |
| Epoch + Group + Epoch  ×  Group |  | 0.20 |  | 0.02 |  | 0.09 |  | 0.85 |  | 1.70 |  |
| Group |  | 0.200 |  | 0.004 |  | 0.02 |  | 4.86 |  | 1.21 |  |
|  | | | | | | | | | | | |
| *Note.*  All models include subject. The Models column indicates the predictors included in each model, the P(M) column the prior model probability, the P(M \| data) column the posterior model probability, the BFM column the posterior model odds, and the BF_01_ column the Bayes factors of all models compared to the null model. The error is an estimate of the numerical error in the computation of the Bayes factor. | | | | | | | | | | | |

| **Supplementary Table 2. Bayesian model comparisons for accuracy learning scores** | | | | | | | | | | | |
| --- | --- | --- | --- | --- | --- | --- | --- | --- | --- | --- | --- |
| **Models** | | **P(M)** | | **P(M\|data)** | | **BF _M_** | | **BF _01_** | | **error %** | |
| Null model (incl. subject) |  | 0.20 |  | 2.52e -7 |  | 1.01e -6 |  | 1.00 |  |  |  |
| Epoch + Group + Epoch  ×  Group |  | 0.20 |  | 0.47 |  | 3.50 |  | 5.40e -7 |  | 2.42 |  |
| Epoch + Group |  | 0.20 |  | 0.33 |  | 1.98 |  | 7.61e -7 |  | 2.73 |  |
| Group |  | 0.20 |  | 0.20 |  | 1.01 |  | 1.25e -6 |  | 2.24 |  |
| Epoch |  | 0.20 |  | 3.81e -7 |  | 1.52e -6 |  | 0.66 |  | 0.47 |  |
|  | | | | | | | | | | | |
| *Note.*  All models include subject. The Models column indicates the predictors included in each model, the P(M) column the prior model probability, the P(M \| data) column the posterior model probability, the BF_M_ column the posterior model odds, and the BF_01_ column the Bayes factors of all models compared to the null model. The error is an estimate of the numerical error in the computation of the Bayes factor. | | | | | | | | | | | |

**Standardized learning scores**

The instructions in the experiment could cause significant differences in the average RTs and accuracies (i.e., all valid trials collapsed together) between the two experimental groups. To ensure that our results on the learning measures were not due to the differences in the average RTs and accuracies, we repeated all our primary analyses with standardized scores. To this end, we divided the learning scores (median RTs for low-probability triplets minus median RTs for high-probability triplets) by the average of RTs for the high- and low-probability triplets of the given epoch, for each participant and each epoch. Similarly, we divided the learning scores (mean accuracy for high-probability triplets minus mean accuracy for low-probability triplets) by the average of accuracies for the high- and low-probability triplets of the given epoch, for each participant and each epoch. RT and accuracy standardized learning scores were analyzed with mixed-design ANOVAs with the within-subject factor of Epoch (Epoch 1 to 4), and the between-subjects factor of Group (Accuracy Group vs. Speed Group).

**Results of the Different Instruction Phase**

***RT measures***

First, we compared the standardized learning scores between the two groups in the Different Instruction Phase. The main effect of Group was not significant, *F*(1, 59) = 0.40, *p* = .53, *η*_p_^2^ = .01, indicating that, in accordance with the results of the non-standardized data, the two groups exhibited similar learning scores in the task. The Bayesian comparison of means also supported the lack of difference, BF_01_ = 4.32. The ANOVA revealed a main effect of Epoch, *F*(2.24, 132.58) = 2.99, *p* = .048, *η*_p_^2^ = .05, suggesting that, in accordance with the non-standardized data, learning scores changed during the task: they became larger from Epoch 1 and Epoch 2 (*p* = .005), but remained unchanged after that (each *p* > .09). The interaction of Epoch and Group did not reach significance, *F*(3, 177) = 1.39, *p* = .25, *η*_p_^2^ = .02, indicating the lack of significant group differences in the dynamics of learning over the epochs.

***Bayesian Model Averaging in the Different Instruction Phase in standardized RT measures***

We conducted a Bayesian repeated-measures ANOVA on the standardized learning scores with the within-subject factor of Epoch (Epoch 1-4) and the between-subjects factor of Group (Accuracy Group vs. Speed Group). The Bayesian ANOVA revealed anecdotal evidence for the inclusion of the Epoch factor, substantial evidence for the exclusion Group factor, and strong evidence for the exclusion of the interaction (Supplementary Table 3). This result suggests that the change of performance was independent of the instructions, and the overall statistical knowledge was not different between groups. The model comparisons can be found in Supplementary Table 4.

| **Supplementary Table 3. Analysis of effects of the standardized RT learning scores** | | | | | | | |
| --- | --- | --- | --- | --- | --- | --- | --- |
| **Effects** | | **P(incl)** | | **P(incl\|data)** | | **BF _exclusion_** | |
| Epoch |  | 0.60 |  | 0.54 |  | 1.27 |  |
| Group |  | 0.60 |  | 0.18 |  | 6.80 |  |
| Epoch  ×  Group |  | 0.20 |  | 0.02 |  | 11.66 |  |
|  | | | | | | | |

Note: The column Effects indicates the main effects and interactions. The P(incl) column denotes the prior, and the P(incl|data) the posterior inclusion probability. The BF_exclusion_ column indicates the change from prior to posterior odds.

| **Supplementary Table 4. Bayesian model comparisons for standardized RT learning scores** | | | | | | | | | | | |
| --- | --- | --- | --- | --- | --- | --- | --- | --- | --- | --- | --- |
| **Models** | | **P(M)** | | **P(M\|data)** | | **BF _M_** | | **BF _01_** | | **error %** | |
| Null model (incl. subject) |  | 0.20 |  | 0.38 |  | 2.49 |  | 1.00 |  |  |  |
| Epoch |  | 0.20 |  | 0.44 |  | 3.09 |  | 0.88 |  | 0.73 |  |
| Epoch + Group |  | 0.20 |  | 0.08 |  | 0.37 |  | 4.55 |  | 1.82 |  |
| Group |  | 0.20 |  | 0.08 |  | 0.33 |  | 5.08 |  | 2.01 |  |
| Epoch + Group + Epoch  ×  Group |  | 0.20 |  | 0.02 |  | 0.09 |  | 18.25 |  | 1.27 |  |
|  | | | | | | | | | | | |
| *Note.*  All models include subject. All models include subject. The Models column indicates the predictors included in each model, the P(M) column the prior model probability, the P(M \| data) column the posterior model probability, the BF_M_ column the posterior model odds, and the BF_01_ column the Bayes factors of all models compared to the null model. The error is an estimate of the numerical error in the computation of the Bayes factor. | | | | | | | | | | | |

***Accuracy measures***

We also compared the standardized accuracy learning scores between the two groups in the Different Instruction Phase. Importantly, the main effect of Group was significant, *F*(1, 59) = 46.17, *p* < .001, *η*_p_^2^ = .44: the Speed Group showed learning in accuracy measures, while the Accuracy Group did not (BF_01_ < 0.001). A significant main effect of Epoch was found, *F*(3, 177) = 5.21, *p* = .002, *η*_p_^2^ = .08. The pairwise comparisons revealed a significant decrease between Epoch 3 and Epoch 4 (*p* = .01). The Epoch × Group interaction was also significant, *F*(3, 177) = 4.82, *p* = .003, *η*_p_^2^ = .08. The pairwise comparisons revealed no change in learning scores in the Accuracy Group (each *p* > .87). On the contrary, in the Speed Group, we found a change in learning scores between Epoch 1 and Epoch 2 (*p* = .02), between Epoch 2 and Epoch 3 (*p* = .01), and between Epoch 3 and 4 (*p* = .001).

***Bayesian Model Averaging in the Different Instruction Phase in standardized accuracy measures***

We ran a Bayesian repeated-measures ANOVA on the standardized accuracy learning scores with the same factors as for the RT analysis. Averaged across all models, the Bayesian ANOVA strongly supported the inclusion of the Group factor, but also the inclusion of the Epoch factor and the Epoch  ×  Group interaction (Supplementary Table 5). The results support that statistical learning in accuracy measures is determined by the instructions, and the learning trajectory is different between groups. The model comparisons can be found in Supplementary Table 6.

| **Supplementary Table 5. Analysis of effects of the standardized accuracy learning scores** | | | | | | | |
| --- | --- | --- | --- | --- | --- | --- | --- |
| **Effects** | | **P(incl)** | | **P(incl\|data)** | | **BF _exclusion_** | |
| Epoch |  | 0.60 |  | 0.99 |  | 0.01 |  |
| Group |  | 0.60 |  | 1.00 |  | 9.87e -8 |  |
| Epoch  ×  Group |  | 0.20 |  | 0.92 |  | 0.02 |  |
|  | | | | | | | |

Note: The column Effects indicates the main effects and interactions. The P(incl) column denotes the prior, and the P(incl|data) the posterior inclusion probability. The BF_exclusion_ column indicates the change from prior from posterior odds.

| **Supplementary Table 6. Bayesian model comparisons for standardized accuracy learning scores** | | | | | | | | | | | |
| --- | --- | --- | --- | --- | --- | --- | --- | --- | --- | --- | --- |
| **Models** | | **P(M)** | | **P(M\|data)** | | **BF _M_** | | **BF _01_** | | **error %** | |
| Null model (incl. subject) |  | 0.20 |  | 7.91e -9 |  | 3.16e -8 |  | 1.00 |  |  |  |
| Epoch + Group + Epoch  ×  Group |  | 0.20 |  | 0.92 |  | 45.95 |  | 8.60e -9 |  | 2.66 |  |
| Epoch + Group |  | 0.20 |  | 0.07 |  | 0.31 |  | 1.12e -7 |  | 0.74 |  |
| Group |  | 0.20 |  | 0.01 |  | 0.04 |  | 8.49e -7 |  | 1.77 |  |
| Epoch |  | 0.20 |  | 5.79e -8 |  | 2.32e -7 |  | 0.14 |  | 0.53 |  |
|  | | | | | | | | | | | |
| *Note.*  All models include subject. All models include subject. The Models column indicates the predictors included in each model, the P(M) column the prior model probability, the P(M \| data) column the posterior model probability, the BF_M_ column the posterior model odds, and the BF_01_ column the Bayes factors of all models compared to the null model. The error is an estimate of the numerical error in the computation of the Bayes factor. | | | | | | | | | | | |

**Results of the Same Instruction Phase**

We compared the acquired knowledge of the two groups in RT measures with standardized learning scores, and no difference was found between groups, *t*(59) = -0.58, *p* = .57, BF_01_ = 4.46. We also compared the acquired knowledge of the two groups in accuracy measures, and we did not find differences between the two groups, *t*(59) = 0.89, *p* = .38, BF_01_ = 3.61. The Bayes factors also support the lack of difference for both comparisons.

**Analyses including the incorrect trials**

We analyzed RTs also with the incorrect trials included. For the analysis of the Different Instruction Phase, we ran a mixed-design ANOVA with the within-subject factors of Triplet (high- vs. low-probability triplets) and Epoch (Epoch 1 to 4) and the between-subject factor of Group (Accuracy Group vs. Speed Group).

**Different Instruction Phase**

The main effect of Group was highly significant, *F*(1, 59) = 58.85, *p* < .001, *η*_p_^2^ = .50, indicating that the Speed Group was faster than the Accuracy Group. The Bayesian comparison of means also favored the difference, BF_01_ < 0.001. The main effect of Epoch was significant, *F*(3,177) = 7.23, *p <* .001, *η*_p_^2^ = .11, indicating a change in RTs over the course of the task. The pairwise comparisons revealed that there was no difference in RTs between Epoch 1 and Epoch 2 (*p* = .41); however, after that, RTs became faster between every consecutive epoch (each *p* < .01). The interaction between the Epoch and Group factors was approaching significance, *F*(1.96, 115.70) = 2.41, *p* = .10, *η*_p_^2^ = .04.

The main effect of Triplet was significant, *F*(1, 59) = 24.04, *p* < .001, *η*_p_^2^ = .29: faster RTs were found for high-probability triplets compared to low-probability triplets (BF_01_ = 0.001). The interaction between Triplet and the Group factors was significant, *F*(1, 59) = 7.46, *p* = .008, *η*_p_^2^ = .11, BF_01_ = 0.20: only the Accuracy Group showed learning (*p* < .001), and learning was not measurable in the Speed Group (*p* = .13) (Supplementary Figure 1). The interaction between the Epoch and Triplet factors was also significant, *F*(3,177) = 5.14, *p* = .002, *η*_p_^2^ = .08: post-hoc comparisons revealed that triplet learning was not detectable in the first epoch (*p* = .35), but in the remaining epochs (*p* < .001 in Epoch 2 and Epoch 3, and trend-level difference in Epoch 4, *p* = .06). The interaction between Epoch, Triplet, and Group was not significant, *F*(80.92, 171.90) = 1.06, *p* = .37, *η*_p_^2^ = .02.

We repeated the analyses with standardized scores. The main effect of Group was significant, *F*(1,59) = 6.47, *p* = .01, *η*_p_^2^ = .10, because learning was higher for the Accuracy Group. The Bayesian comparison of means also supported the difference between groups, BF_01_ = 0.31. The main effect of Epoch was significant, *F*(3,177) = 3.21, *p* = .02, *η*_p_^2^ = .05, indicating a difference in statistical learning between epochs. The pairwise comparisons revealed a difference between Epoch 1 and Epoch 2 (*p* = .01), but not between other two consecutive epochs (each *p* > .33). The interaction between Epoch and Group was not significant, *F*(3,177) = 0.39, *p* = .76, *η*_p_^2^ = .01, indicating that statistical learning was not changing differently between the two groups over the course of the session.


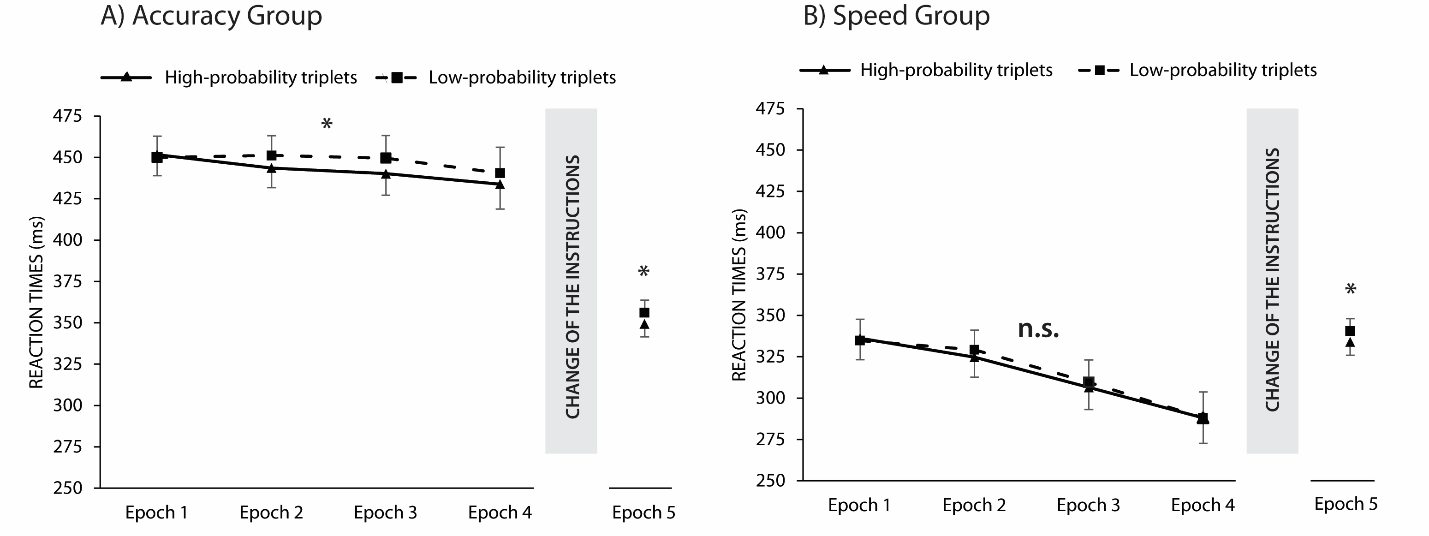


**Supplementary Figure 1. Performance in the (A) Accuracy Group and (B) Speed Group when incorrect responses were also considered.** The horizontal axis shows the five epochs of the task and the vertical axis the RTs. The solid line represents the RTs for the high-probability triplets, and the dashed line indicates the RTs for the low-probability triplets. The error bars denote the standard error of the mean (SEM). Please note the gap between the two lines indicates the learning of statistical regularities. In the Same Instruction Phase (first four epochs), RTs for high- and low-probability triplets only differed in the Accuracy Group. However, a difference between the two trial types was measurable in both groups after the change of the instructions. * p < .05

**Same Instruction Phase**

For the analysis of the Same Instruction Phase, we ran a mixed-design ANOVA with the within-subject factor of Triplet (high- vs. low-probability triplets) and the between-subjects factor of Group (Accuracy Group vs. Speed Group).

The main effect of Group was not significant, *F*(1, 59) = 2.00, *p* = .16, *η*_p_^2^ = .03, indicating that the speed difference disappeared after the change of the instructions; however, according to Bayesian comparison of the group means, the lack of difference was only anecdotal, BF_01_ = 2.19. The main effect of Triplet was significant, *F*(1, 59) = 41.08 *p* < .001, *η*_p_^2^ = .41: faster RTs were found for high-probability triplets compared to low-probability triplets (BF_01_ < 0.001). The interaction between Triplet and the Group factors was non-significant, *F*(1, 59) = 0.02, *p* = .90, *η*_p_^2^ < .001: despite the differences in the Different Instruction Phase, a comparable level of statistical knowledge was revealed after the change of the instructions (*p* = .90). The Bayesian comparison of the difference also supported the null-hypothesis, BF_01_ = 5.15 (Supplementary Figure 2). We repeated the analyses with the standardized scores and again, no difference was found between groups, *t*(59) = -0.07, *p* = .95, BF_01_ = 5.17.


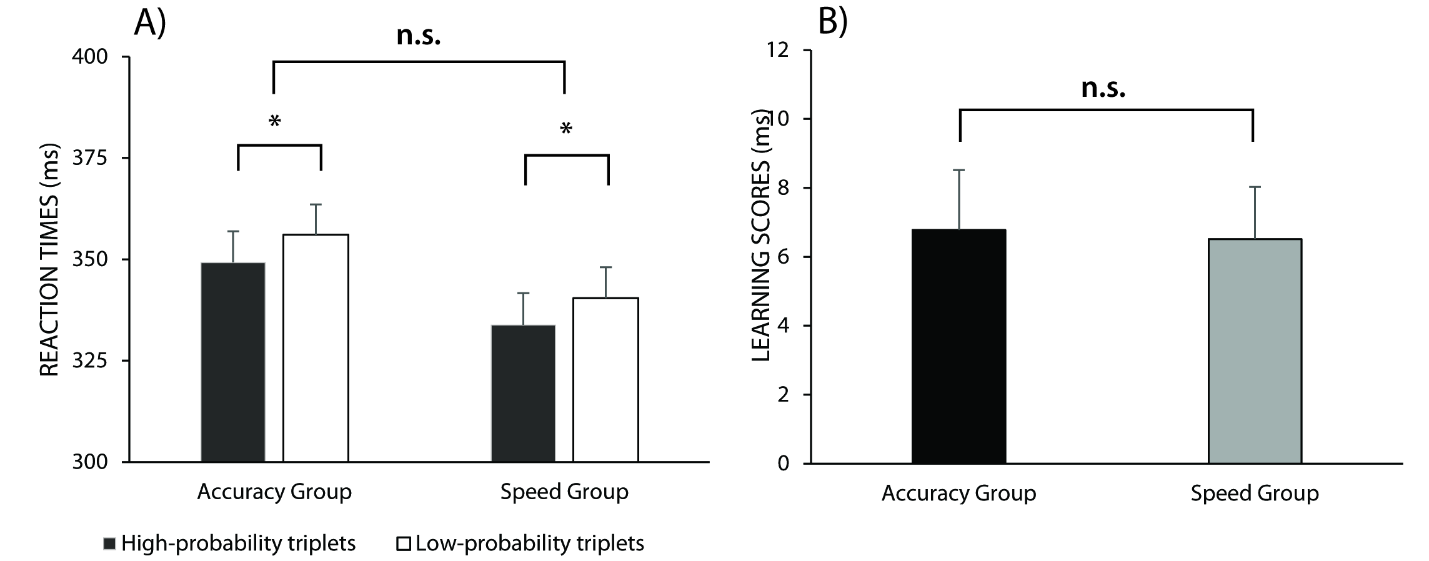


**Supplementary Figure 2. Comparison of the high- and low-probability triplets (A) and learning scores (B) in the Same Instruction Phase (including incorrect responses).** The vertical axis indicates the RTs (A) or the learning scores (RTs for low-probability triplets minus RTs for high-probability triplets, B), and the horizontal axis represents the two groups. The error bars denote the SEM. Although statistical knowledge was detected, no significant difference was found between groups, and the lack of difference was confirmed by Bayesian analysis. * *p* < .05

***Bayesian Model Averaging in the Different Instruction Phase with the incorrect responses included***

We conducted a Bayesian ANOVA on the difference between the two levels of the Triplet factor, (i.e., high-probability and low-probability triplets) with the within-subject factor of Epoch (Epoch 1-4) and the between-subject factor of Group (Accuracy Group vs. Speed Group). The Bayesian ANOVA supported the inclusion of the Epoch factor (Supplementary Table 7). The model tended to favor the exclusion of the interaction, and provided anecdotal evidence for the inclusion of the Group factor. This result suggests that the learning scores changed throughout the task, and this change was independent of the instructions (see detailed model comparisons in Supplementary Table 8).

| **Supplementary Table 7. Analysis of effects of the RT learning scores including errors** | | | | | | | |
| --- | --- | --- | --- | --- | --- | --- | --- |
| **Effects** | | **P(incl)** | | **P(incl\|data)** | | **BF _excl_** | |
| Epoch |  | 0.60 |  | 0.97 |  | 0.05 |  |
| Group |  | 0.60 |  | 0.64 |  | 0.85 |  |
| Epoch  ×  Group |  | 0.20 |  | 0.08 |  | 2.73 |  |
|  | | | | | | | |

Note: The column Effects indicates the main effects and interactions. The P(incl) column denotes the prior, and the P(incl|data) the posterior inclusion probability. The BF_exclusion_ column indicates the change from prior to posterior odds.

| **Supplementary Table 8. Bayesian model comparisons for RT learning scores including the errors** | | | | | | | | | | | |
| --- | --- | --- | --- | --- | --- | --- | --- | --- | --- | --- | --- |
| **Models** | | **P(M)** | | **P(M\|data)** | | **BF _M_** | | **BF _01_** | | **error %** | |
| Null model (incl. subject) |  | 0.20 |  | 0.01 |  | 0.05 |  | 1.00 |  |  |  |
| Epoch + Group |  | 0.20 |  | 0.54 |  | 4.69 |  | 0.02 |  | 9.78 |  |
| Epoch |  | 0.20 |  | 0.35 |  | 2.13 |  | 0.04 |  | 1.26 |  |
| Epoch + Group + Epoch  ×  Group |  | 0.20 |  | 0.08 |  | 0.37 |  | 0.15 |  | 3.72 |  |
| Group |  | 0.20 |  | 0.02 |  | 0.07 |  | 0.78 |  | 3.37 |  |
|  | | | | | | | | | | | |
| *Note.*  All models include subject. All models include subject. The Models column indicates the predictors included in each model, the P(M) column the prior model probability, the P(M \| data) column the posterior model probability, the BF_M_ column the posterior model odds, and the BF_01_ column the Bayes factors of all models compared to the null model. The error is an estimate of the numerical error in the computation of the Bayes factor. | | | | | | | | | | | |

**References**

Ellis PD. 2010. The Essential Guide to Effect Sizes, The Essential Guide to Effect Sizes.

Faul F, Erdfelder E, Lang AG, Buchner A. 2007. G*Power 3: A flexible statistical power analysis program for the social, behavioral, and biomedical sciences. In: Behavior Research Methods.

Nemeth D, Janacsek K, Fiser J. 2013. Age-dependent and coordinated shift in performance between implicit and explicit skill learning. Front Comput Neurosci. 7:147.

Nemeth D, Janacsek K, Király K, Londe Z, Németh K, Fazekas K, Adam I, Király E, Csányi A. 2013. Probabilistic sequence learning in mild cognitive impairment. Front Hum Neurosci. 7:318.

Nemeth D, Janacsek K, Polner B, Kovacs ZA. 2013. Boosting human learning by hypnosis. Cereb Cortex. 23:801–805.

Virag M, Janacsek K, Horvath A, Bujdoso Z, Fabo D, Nemeth D. 2015. Competition between frontal lobe functions and implicit sequence learning: evidence from the long-term effects of alcohol. Exp Brain Res. 233:2081–2089.
